# NeoMutate: an ensemble machine learning framework for the prediction of somatic mutations in cancer

**DOI:** 10.1186/s12920-019-0508-5

**Published:** 2019-05-16

**Authors:** Irantzu Anzar, Angelina Sverchkova, Richard Stratford, Trevor Clancy

**Affiliations:** OncoImmunity AS, Oslo Cancer Cluster, Ullernchausseen 64/66, 0379 Oslo, Norway

**Keywords:** Somatic variant detection, Machine learning, Cancer genomics, Precision medicine

## Abstract

**Background:**

The accurate screening of tumor genomic landscapes for somatic mutations using high-throughput sequencing involves a crucial step in precise clinical diagnosis and targeted therapy. However, the complex inherent features of cancer tissue, especially, tumor genetic intra-heterogeneity coupled with the problem of sequencing and alignment artifacts, makes somatic variant calling a challenging task. Current variant filtering strategies, such as rule-based filtering and consensus voting of different algorithms, have previously helped to increase specificity, although comes at the cost of sensitivity.

**Methods:**

In light of this, we have developed the NeoMutate framework which incorporates 7 supervised machine learning (ML) algorithms to exploit the strengths of multiple variant callers, using a non-redundant set of biological and sequence features. We benchmarked NeoMutate by simulating more than 10,000 bona fide cancer-related mutations into three well-characterized Genome in a Bottle (GIAB) reference samples.

**Results:**

A robust and exhaustive evaluation of NeoMutate’s performance based on 5-fold cross validation experiments, in addition to 3 independent tests, demonstrated a substantially improved variant detection accuracy compared to any of its individual composite variant callers and consensus calling of multiple tools.

**Conclusions:**

We show here that integrating multiple tools in an ensemble ML layer optimizes somatic variant detection rates, leading to a potentially improved variant selection framework for the diagnosis and treatment of cancer.

**Electronic supplementary material:**

The online version of this article (10.1186/s12920-019-0508-5) contains supplementary material, which is available to authorized users.

## Introduction

One of the hallmarks of cancer is the accumulation of somatic genomic alterations, acquired during a cell’s life cycle and development [1]. Throughout cancer progression, some of these variants may dictate clinical response to therapy [2]. The reliable detection of mutations in cancer genomes is therefore key to our understanding of the genomic basis of tumorigenesis and patient survival. Unfortunately, the accurate prediction of somatic variants from tumor and matched normal samples using next generation sequencing (NGS) data remains challenging [3–5]. The complex features of tumor tissue, particularly intra-tumor heterogeneity and structural rearrangements, in addition to normal tissue admixtures, intrinsic sequencing errors and mapping ambiguities, are sources of noise and complexity in the analysis. As a result, the current somatic variant calling strategies generate high false prediction rates [4].

### Background to the main individual variant callers: strengths and weaknesses

The advent of NGS technologies has enabled the comprehensive detection of de novo DNA aberrations in a tumor. The continued advancements in variant detection in the last decade has resulted in a wide collection of bioinformatics tools based on different underlying statistical models. However, their results vary widely as demonstrated in several benchmarking studies [3–11] and none have emerged as a gold standard and been universally adopted by the field. The specific mathematical algorithms operating each variant caller plus the enormous experimental and biological variability in tumor samples creates ambiguities in performance across different datasets. One of the biggest challenges is to accurately discriminate between low allele frequencies of true somatic variants (particularly relevant in impure samples or heterogeneous tumors) and sequencing or alignment artifacts.

VarScan2 [12] and VarDict [13], apply a heuristic methodology followed by a Fisher’s test to increase the reliability of the detected somatic variants. An appropriate parameter fine-tuning and threshold selection in heuristic tools can achieve a good performance in low-frequency variant detection; unfortunately optimal parameter values are frequently unknown and dependent on the specific dataset [14]. SomaticSniper [15], based on a joint genotype analysis, is designed for single nucleotide variants (SNVs) detection on low-coverage data. However, low-depth coupled with the underlying diploid assumption in tumor genomes, means that the method is not optimal for detecting of low frequency variants. Modeling joint allele frequencies instead of joint genotypes, as done by Strelka [16], is a straightforward way to call low-frequency variants. Strelka2 [17], a variant calling method which builds upon its predecessor Strelka, applies an additional random forest based variant re-scoring step to improve accuracy on liquid and late-stage tumours. Finally, methods like MuTect2 (available in GATK > 3.5) [18], Freebayes [19] and Lancet [20] follow a haplotype-based strategy, where the reads are locally assembled representing the candidate haplotypes in De Bruijn graphs, aiding the detection of co-occurring variants.

Unfortunately, identifying the most appropriate variant calling tool for each scenario, in addition to the adequate fine-tuning of its parameters for optimal performance, is a complex and time-consuming task [3–9], which complicates the ability to make rational clinical decisions based on called variants in an actionable timeframe [3].

### The effect of integrating variant callers: their union or intersection

Due to the low concordance between individual tools, it is natural to address the challenge of somatic variant calling through the integration of two or more tools. Integrating the results of two or three somatic variant callers in a consensus voting filtering strategy has emerged as a standard protocol in many recent studies [21–24] . However, although these consensus calling approaches may help to increase the calling of specific candidates, this often comes at the cost of sensitivity when candidates are identified using the intersection between component tools, and can introduce enormous amount of false positives when they are identified using the union between component tools. Consensus calling may lead to the loss of valuable true positive variants, particularly those that are present at low variant allelic frequencies (VAF). Recent studies have shown that some clinically relevant mutations are miscalled using intersection based approach, such as treatment-induced secondary mutations [25], and thus the intersection of tools is arguably a sub-optimal solution.

### The emergence of machine learning approaches

A relatively recent progression in the variant calling field to address these aforementioned problems is the application of supervised machine learning (ML). In the ML approaches, variant calling can be reduced to a classification problem, whereby each considered genomic position is labeled with its mutation status. Those base pairs that differ from the reference genome in the tumor sample, and are not present in the normal sample, will be subsequently labelled as somatic mutations.

Variant calling strategies that take advantage of supervised ML can be classified into two main categories: (1) variant calling algorithms directly based on ML and (2) ensemble ML approaches that integrate multiple variant callers. SNooPer [26], MutationSeq [27], TNscope [28] and DeepVariant [29] are examples of the former. SNooPer is based on a random forest (RF) classifier primarily focused on low-depth sequencing data, resulting in a suboptimal method for low VAF detection. Moreover, SNooPer’s training dataset is based on the GATK HaplotypeCaller [30], which is not well suited to extreme allele frequencies and therefore not recommended for somatic (cancer) variant discovery. The MutationSeq study involved building a feature-based classifier to provide guidelines based on a comparison of four ML algorithms applied to 106 manually selected features, and only focuses on SNVs. Many of the 106 selected features are redundant and used by several variant callers themselves, however in one sense, this indicated an emergence toward ensemble ML to address the problem of selecting arbitrary thresholds by each individual tool for selected features. TNScope, like Mutect2, is haplotype-based variant caller that applies rule-based approach by default. TNScope also provides a ML model trained using a RF from the Genome in a Bottle (GIAB) data [31]. DeepVariant uses labeled true genotypes if and when they are available and has the goal of generalizing across sequencing technologies, genome builds, and experimental designs. It applies deep learning using convolutional neural networks to achieve high sensitivity and low specificity, and has not been developed or tested specifically for somatic variant calling.

In the category of ensemble based variant callers it is first worth mentioning BAYSIC [32]. While BAYSIC uses an unsupervised learning approach based on Bayesian statistics, it does also attempt to integrate four variant callers in a common framework. It is however restricted to scale by the need to intimately understand the estimated error rates of each individual tool, as it produces poster probabilities based on these estimates. Additionally, BAYSIC comes with the limitation that arbitrary thresholds depending on the tolerance for sensitivity versus specificity predetermine its performance. SomaticSeq [33] and SMuRF [34] are tools that use the ensemble of several variant callers in a supervised ML approach. SMuRF, although trained on the International Cancer Genome Consortium (ICGC) community-curated data that is not simulated, it is restricted to a limited amount of training data from only two tumors (both of them having high tumor purity (cancer-cell fraction > 0.92)) and focused on whole genome sequencing data. SomaticSeq integrates five third-party variant callers through the use of > 70 features into an ensemble of decision trees. Unfortunately, as with MutationSeq, these large number features are somewhat redundant, subject to arbitrary thresholds, and variation between the individual tools and VCF formats, dramatically affecting the portability of the tool. These limitations may also affect the scalability of such approaches and thereby the subsequent benefits that can be reaped from integrating multiple callers in ensemble ML framework.

Following the concept of aggregating the results from multiple variant calling algorithms in an ensemble ML layer, to yield an overall improved performance compared to the individual component tools or consensus of tools, we have developed NeoMutate. In this study, NeoMutate combines the relative sensitivities of 7 state-of-the-art variant calling tools, including MuTect2 [18], Strelka2 [17], SomaticSniper [15], VarScan2 [12], VarDict [13], Lancet [20], Freebayes [19], in order to gain improved performance. NeoMutate extracts 17 core non-redundant biological and sequencing features from the aligned BAM files. These features, for each candidate site, are incorporated with only three of the essential variant annotations provided by the individual tools. This design facilitates the scalability of the solution to in principle encompass an unlimited number of variant callers. Unlike the previous two ensemble based supervised ML approaches (SMuRF and SomaticSeq), a comprehensive survey of ML classifiers is profiled (including logistic regression, support vector machines, Gaussian Naïve Bayes, random forests, gradient boosting decision trees and neural networks). All of the ensemble ML models in NeoMutate were trained with more than 3000 bona fide cancer variants from Catalogue of Somatic Mutations in Cancer (COSMIC) [35]. Standard 5-fold cross validation and independent tests demonstrated an improved variant detection accuracy compared to the individual tools and consensus voting strategies. In particular, decision-tree type models stood out as having surpassed the standard filtering approaches and individual tools. Using a range of metrics, we demonstrate how the NeoMutate framework optimizes simultaneously for sensitivity and specificity. This is achieved using the minimal required, albeit precise features that are most relevant to accurate variant calling, rendering NeoMutate with a high degree of scalability and portability.

## Methods

### Generation of synthetic tumor variant datasets and NGS data processing

**Fig. 1:**
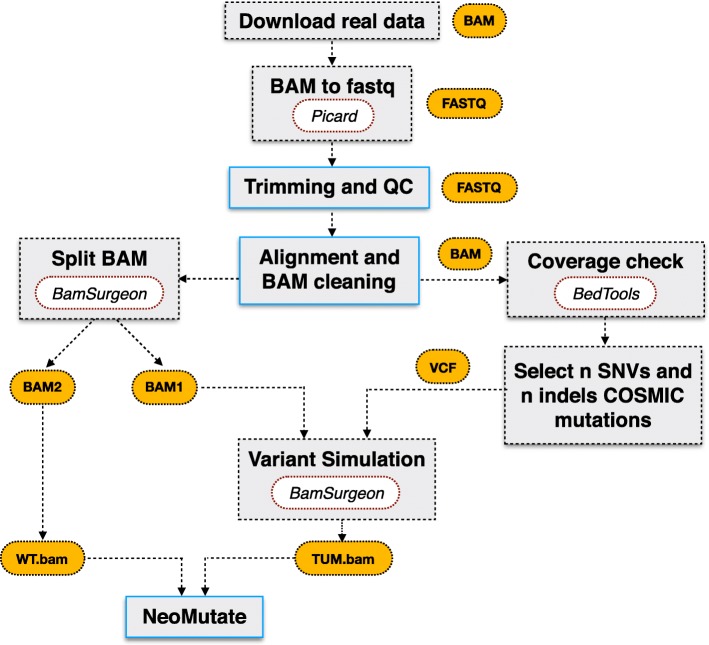
In silico variant simulation workflow on real data using BamSurgeon: NA12878, NA12891 and NA24631 real datasets were spiked-in with non-overlapping variant subsets at different allele frequencies (ranging from 0.01 to 1) extracted from COSMIC database for S1, S2 and S3 simulation experiments. An additional simulation was performed using NA24631 dataset and non-COSMIC random mutations having VAF < =0.2. Text boxes coloured with blue borders represent steps embedded in NeoMutate workflow

Following the methodology used by ICGC-TCGA DREAM Somatic Mutation Calling Challenge for the synthetic dataset generation, BamSurgeon was used to spike mutations into three different well-known datasets included in the 1000 Genome Project (Table 1). The three WES sample files were downloaded in BAM format. In order to test all the functionalities of the NeoMutate workflow, the BAM files were converted back to fastq format using Picard SamToFastq (2.6.0) utility [36] (Fig. 1). Fastq files were then processed using NeoMutate, including data quality control, adapter clipping, alignment and alignment post-processing. Quality assessment of the fastq files was checked using FastQC (v0.11.5) [37] and subsequently, adapter clipping, artefact removal, quality trimming through BBduk (37.50) [38] was conducted when required. The high quality paired-end reads were aligned to the human hs37d5 reference genome, which included data from GRCh37, the rCRS mitochondrial sequence, Human herpesvirus 4 type 1 and the concatenated decoy sequences, leading to a better mapping quality. Alignment was performed using BWA-MEM (0.7.17-r1188) [39], based on the Burrows-Wheeler transformation. The resulting alignment files (BAM) were cleaned following the recommended standardized GATK practices [40], including duplicate marking, BQSR and Indel realignment. To check the quality of the raw and intermediate BAM files produced at each post-alignment step, several metrics were collected and measured using SAMtools (1.5) [41], such as: number of reads mapped/unmapped, average length, mismatches (see Additional file 1: Table S1, for a detailed overview of collected metrics).

**Table 1:** List of GIAB datasets used for NeoMutate benchmarking. The data was downloaded in BAM format and converted back to fastq in order to fully test all the functionalities of the workflow. (WES: whole-exome sequencing; PE: paired-end)

Sample ID	Lab	Project	Library type	Read length	Insert size	File format	Number of reads before trimming	Number of reads after trimming	% kept reads
NA12878	Broad Institute	CEU Trio Analysis (son)	WES, PE	76 bp	155 bp	BAM	118,969,048	89,151,231	74.94
NA12891	Broad Institute	CEU Trio Analysis (father)	WES, PE	76 bp	155 bp	BAM	116,639,621	88,079,244	75.51
NA24631	Oslo University Hospital	Asian (Han chinese) Trio (son)	WES, PE	125 bp	202 bp	BAM	61,001,625	60,852,682	99.76

The analysis-ready BAM files, one per sample, were randomly sampled into two non-overlapping subsets of equal size for NA12878 and NA12891, and in 0.4/0.6 ratio for the NA24631 in order to slightly increase variability (Fig. 1) (Additional file 1: Table S2). Four different simulations, S1, S2, S3 and S4 (Additional file 2 S1 - S4) were performed using the three genomes and non-overlapping spectrum of synthetic mutations for an exhaustive benchmark of NeoMutate (Table 2). After checking coverage using BEDTOOLS program (v2.26.0) [42], covered cancer related variants were randomly selected from COSMIC database (v83) [35] for S1, S2 and S3 simulations, including SNVs and indels, and added to one of the sampled sub-BAMs using BAMSurgeon forming the tumor bam file. The VAFs of the synthetic added mutation ranged from 0.01 to 1, allowing simulation of multiple subclones or sample contamination. The S4 simulation was implemented using variants not present in COSMIC dataset, but those generated at random positions in the genome to avoid the bias of some third-party tools towards COSMIC related mutations. Moreover, a more stringent VAF range was chosen for S4, having all the variants VAF < =0.2 (730 of them falling in the category of low allele frequency variants (VAF < =0.05)), in order to increase the complexity of the analysis (see Additional file 1: Table S3, for synthetic mutation spectrum VAF detailed overview). It is important to mention that due to the internal considerations in BAMSurgeon, not all chosen spike-in variants were successfully added probably because their location on low depth genomic regions or their extremely low VAF (Additional file 1: Table S4). However, the overall successful rate for spike-in variants was higher than 0.9 for most of the cases, as those variants were considered as ground truth for evaluating the models performance.

**Table 2:** Simulation experiments design overview

Simulation ID	Sample ID	is_COSMIC	VAF range	All	SNV	Insertions	Deletions
S1	NA12878	yes	0.01–1	3600	3000	300	300
S2	NA12891	yes	0.01–1	6000	5000	500	500
S3	NA24631	yes	0.01–1	6000	4000	1000	1000
S4	NA24631	no	0.01–0.2	5000	3000	1012	988

### Ensemble variant calling

NeoMutate, in this study, incorporated an ensemble of 7 different state-of-the art tools, including: MuTect2, Strelka2, VarScan2, VarDict, SomaticSniper, Freebayes and Lancet (Table 3), which were executed having as input the tumor-normal paired BAM files with the embedded synthetic mutations. Nearly all default parameters suggested by the developer of each caller were used for running the tools, these are generally considered to be the optimal settings for most data types. (Additional file 1: Table S5). Each somatic caller implements its own assumptions and has specific statistical models to exclude true genetic variations from background noise, which are optimal for certain datatypes but generate worse results for others. Integrating the different algorithms and selecting the union call-set seems a sensible approach for raising sensitivity and adaptability to the different tumor scenarios, the main purpose of this ensemble approach.

**Table 3:** List of individual somatic variant callers embedded in NeoMutate

Tool	Version	Methodology
MuTect2	3.8	Bayesian classifier
Strelka2	2.8.4	Bayesian model of admixture
VarScan2	2.4.3	Heuristic methodology with statistical test
VarDict	1.4	Combined heuristic and statistical algorithm
SomaticSniper	1.0.5.0	Bayesian approach for estimating genotype probabilities
Freebayes	0.1.2	Bayesian model with error probabilities
Lancet	1.0.5	Colored de Bruijn graphs

### Feature selection

Several sequencing and biological features were extracted at the BAM and VCF level in order to collect as much non-redundant information as possible for each variant candidate (Table 4). The bam-readcount program (0.8.0) [43] was called to query and retrieve the requested metrics directly from the BAM files. The features were classified into four distinct groups depending on the carried information (detailed information about each specific feature is provided in Table 4). Each variant site reported by at least one of the tools in the ensemble variant calling (i.e. the union of the 7 algorithms) was coupled with the different set of features, in addition to each tool call status (detected or non-detected). Using BAMSurgeon ground truth VCF file, each candidate site was categorized with the proper somatic status label (“somatic” or “non-somatic”), forming the adequate input data for training the supervised machine learning classifiers. To prevent over-fitting on training data, those features represented as continuous variables were normalized using MinMaxScaler module available in sklearn python library, shrinking their range between 0 and 1.

**Table 4:** List of biological and sequencing features selected for downstream ML analysis

Feature name	Source	Group	Description
indel_or_snp	BAM	3	Is the given variant a SNP, insertion or deletion?
ts_or_tv	BAM	3	Transition or transversion
depth_TUM	BAM	1	Coverage in tumor sample for the given variant position
alt_counts_TUM	BAM	1	Alternative read counts (number of reads supporting the variant)
alt_avg_MQ_TUM	BAM	2	Average mapping quality of reads containing the variant. Quantification of the probability that a read is misplaced.
alt_avg_BQ_TUM	BAM	2	Average base quality of the reads containing the variant. Accuracy of a base sequenced by the sequencing machine.
alt_plus_TUM	BAM	1	Number of reads on the plus/forward strand supporting the variant
alt_minus_TUM	BAM	1	Number of reads on the minus/reverse strand supporting the variant
ref_plus_TUM	BAM	1	Number of reads on the plus/forward strand supporting the reference allele
ref_minus_TUM	BAM	1	Number of reads on the minus/reverse strand supporting the reference allele
VAF	BAM	1	Variant allele frequency
depth_WT	BAM	1	Coverage in normal sample for the given variant position
alt_counts_WT	BAM	1	Number of reads supporting the variant in normal sample (germline risk)
ref_counts_WT	BAM	1	Number of reads supporting the reference in normal sample
num_of_indels_closeby	BAM	3	Are there indels closeby? (false positive risk factor)
GC_content	BAM	3	Number of GC bases relative to the total number of bases located + − 20 bp for the given variant position
shannon_entropy	BAM	3	A mathematical measure of the degree of randomness in a set of data. The smaller the entropy value, the less complex the sequence is.
detection_status	VCF	4	Classification status (“somatic” or “non somatic”) for the given variant caller
“Tool”_F	VCF	4	Quality tag in FILTER column (“PASS” or “non PASS”)
“Tool”_alt_counts	VCF	1, 4	Number of reads supporting the variant reported by the specific tool
“Tool”_ref_counts	VCF	1, 4	Number of reads supporting the reference reported by the specific tool

The ensemble variant calling along with the feature set served as input to the 7 supervised machine learning classifiers: Logistic Regression (LR), Support Vector Machine with linear kernel (SVMl), Gaussian Naïve Bayes (GNB), Decision Tree (DT), Random Forest (RF), Gradient Boosting Decision Tree (GBDT) and Neural Network (NN).

### Variant evaluation by ensemble machine learning framework

Classifiers were comprehensively evaluated in CV experiments and on independent tests with labelled data through a robust and exhaustive set of performance measurements (Table 5). The area under the curve (AUC) of a receiver operating characteristic (ROC) and precision-recall (PR) curves was calculated using the predicted probabilities for each candidate site. PR curves were chosen as they are known to be more informative than ROC curves when the class distribution of the data is unbalanced [44], as is common in somatic variant calling.

**Table 5:** Definition of selected performance metrics used for algorithm evaluation. The four variables present in a 2 × 2 contingency table: true positive (TP) (variants predicted and validated), true negative (TN) (variants not predicted and not validated), false positive (FP) (variants predicted but failed in validation), and false negative (FN) (variants not predicted but validated) are used to calculate the metrics and assess model performance

Metric	Formula	Definition
Accuracy	$\frac{TP+ TN}{\left( TP+ TN+ FP+ FN\right)}$	The ratio of correct calls out of the total number of positions.
Precision	$\frac{TP}{\left( TP+ FP\right)}$	The ratio of correct variant calls out of the total number of variant calls. Synonyms: Positive predictive value (PPV)
Recall	$\frac{TP}{\left( TP+ FN\right)}$	The ratio of correct variant calls out of the total number of variant positions. Synonyms: Sensitivity, true-positive rate (TPR).
False discovery rate (FDR)	$\frac{FP}{\left( TP+ FP\right)}$	The ratio of incorrect calls out of the total number of variant calls.
F1-Score	$\frac{2\ast Precision\ast Recall}{\left( Precision+ Recall\right)}$	Harmonic mean of precision and recall, where 1 is the best score and 0 the worst. Synonyms: F-score
Matthews correlation coefficient (MCC)	$\frac{TP\ast TN- FP\ast FN}{\sqrt{\left( TP+ FP\left)\right( TP+ FN\right)\left( TN+ FP\right)\left( TN+ FN\right)}}$	A measure of the quality of binary (two-class) classifications. The MCC represents the correlation coefficient between the observed and predicted binary classifications, where −1 indicates a completely wrong binary classifier while 1 indicates a completely correct classifier.

## Results

### The NeoMutate workflow for robust and scalable ML based ensemble variant calling

**Fig. 2:**
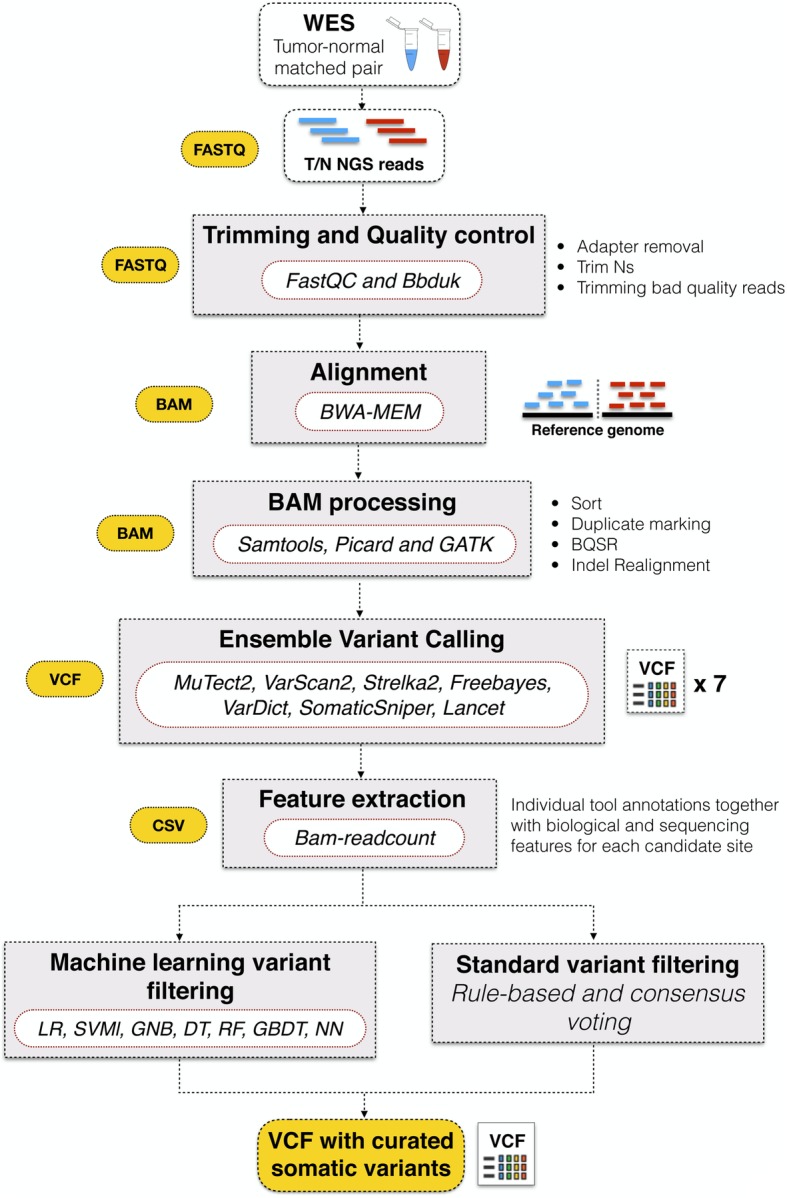
NeoMutate workflow: This figure illustrates the main steps executed during NeoMutate framework, where raw reads from nearly any sequencing technology platform are transformed into an accurate list of prioritized somatic variants. Its modular architecture consists in quality control of the raw data, alignment and BAM post-processing, ensemble variant calling and machine learning boosted variant filtering step. 7 machine learning models are trained using the ensemble calling plus a set of biological and sequencing relevant features. Each algorithm will provide a mutational status classification per variant yielding a high-confidence somatic mutation call set

NeoMutate attempts to address the inherent complexities of somatic variant calling in cancer by applying an ensemble based supervised ML approach to improve performance. Full details of NeoMutate’s methodology are described in the methods section. NeoMutate integrates different variant calling tools in an all-inclusive modular pipeline to capture a more comprehensive mutational profile and performs an ensemble ML based step for boosting detection accuracy (Fig. 2). The workflow can be divided in two main components: (1) an ensemble variant calling pipeline and (2) an ensemble machine learning framework. The former follows the best practices of the genome analysis toolkit (GATK) [40] in a high-performance robust automated workflow, which consist of data preprocessing, alignment, post-alignment processing, followed by the integration of 7 variant calling algorithms. For the purpose of this study, the high-quality processed BAM files are provided as input into 7 state-of-the-art somatic variant calling tools (Table 3). However, it is worth noting that NeoMutate’s modular architecture confers a high flexibility and adaptability to the incorporation of any number of variant calling tools. NeoMutate leverages the strengths of each independent somatic variant calling tool with the aim of increasing sensitivity while maintaining accuracy and capture the full mutational profile from the sequencing data of tumor-normal pairs. The ML based step begins with the annotation of each candidate variant included in the combined set of calls of from the entire ensemble of variant calling with additional genomic and sequencing features. There were 17 non-redundant features extracted from the BAM files and four features extracted from each VCF file. The selected features can be categorized in four groups: 1) read depth, strand bias and allele frequency, 2) base and mapping qualities, 3) variant type and genomic context, and 4) detection status for each caller. The combined individual variant caller annotations together with the additional features are given as input to train the 7 models available in NeoMutate’s ML framework (logistic regression classifier (LRC); support vector machine classifier with linear kernel (SVMl); decision tree (DT); Gaussian Naïve Bayes (GNB); random forest classifier (RFC); gradient boosting decision tree (GBDT); neural network (NN)), to attempt to capture and infer the interplays of features in order to reduce false positive predictions without discarding true events.

### A comprehensive profiling of ML classifiers demonstrates improved performance over standard approaches for variant detection

In order to simulate synthetic ground truth somatic variants, we spiked in bona fide variants from the COSMIC database [35] using BAMSurgeon [45], into three well studied genomes from the GIAB Consortium (NA12878, NA12891 and NA24631). Four different simulations (S1- S4) were conducted to probe effectiveness of the ensemble ML based approaches. A total number of 3421, 5573, 5763 randomly selected COSMIC variants (SNVs and indels, VAF range 0.01–1) were successfully added into NA12878, NA12891 and NA24631 samples, for the S1, S2 and S3 simulations, respectively. Additionally, 4894 non-COSMIC variants (<= 0.2 VAF) were spiked into NA24631 dataset for the S4 simulation. The BAMSurgeon ground truth variants allowed us to assign labels (“somatic” or “non-somatic”) to each candidate site reported in the final call sets for each experiment, allowing for a correct evaluation of the performance. Each candidate mutation was tagged with the validation status determined by the ground truth, in addition to a call status (detected or not detected) for each variant calling tool, classifying them as true-positive or false-positive.

NeoMutate’s performance on somatic variant detection was first evaluated through 5-fold cross-validation (CV) experiments using the reported call set from the S1 simulation experiment. The combined call set from all 7 callers consisted of 12,153 variants, 3235 (26.62%) of which were ground truth somatic mutations (see Additional file 3 for individual and combined tool results). Each candidate variant was annotated with the ground truth status, in addition to the status of each variant calling tool and additional genomic features.

**Fig. 3:**
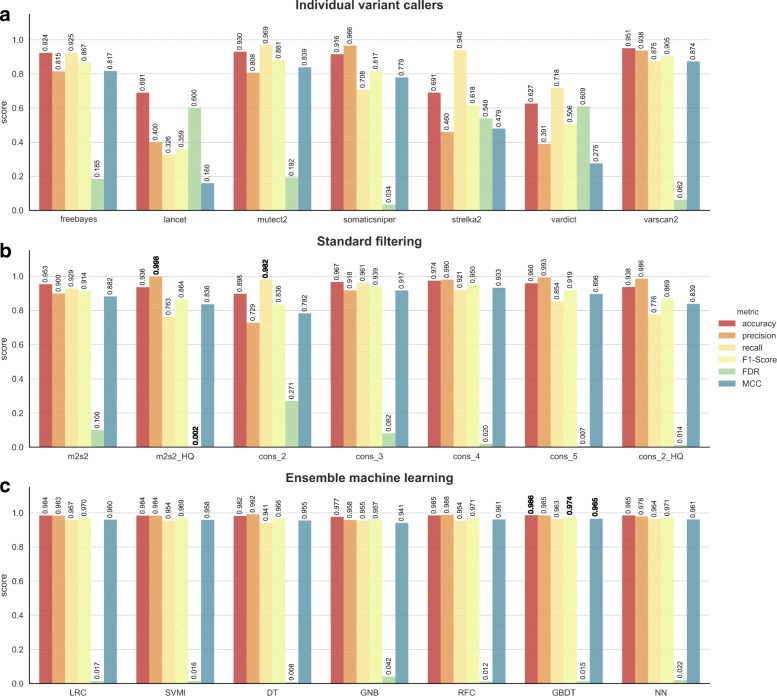
Comprehensive performance evaluation of different approaches. Only those approaches having a sensible recall (> 0.5) were chosen for the comparison. **a**) Individual variant callers raw results evaluation. **b**) Standard filtering results evaluation. *m2 s2*: mutect2 and strelka2 calls intersection; *m2s2_HQ*: mutect2 and strelka2 HQ (only variants tagged as `PASS`) calls intersection; *cons_n*: consensus voting (intersection) of at least n tools; *cons_2_HQ*: consensus voting of the HQ call sets of least 2 tools. **b**) ML results evaluation. *LRC*: logistic regression classifier; *SVMl*: support vector machine classifier with linear kernel; *DT*: decision tree; *GNB*: Gaussian Naïve Bayes; *RFC*: random forest classifier; *GBDT*: gradient boosting decision tree; *NN*: neural network

The performance of all 7 ML models was compared against commonly used standard filtering approaches (Additional file 1: Table S6), in addition to the results from the individual variant callers. Systematic evaluation of the results was performed using a comprehensive set of performance metrics derived from contingency tables that captured the relationship between the prediction status assigned by the method and labels from the ground truth data (Fig. 3, and Additional file 2: Table S1). The winning method for each considered metric is highlighted in bold in Fig. 3. The most stringent considered standard filtering approach, consisting of the intersection between MuTect2 and Strelka2 high quality variants (tagged as PASS in their correspondent VCF files), achieved the highest precision and therefore the lowest FDR. This approach removes a high degree of false positives (FP) at the cost of true positives (TP). The somatic variant calls reported by at least any two callers, won the performance for recall, at the expense of poorer precision. The ML models accomplished the highest balance retrieved TP and FP, as shown in the F1-Score and Mathews Correlation Coefficient (MCC) in Fig. 3 and Additional file 2: Table S1. While the rule-based and consensus voting approaches struggle to balance recall and precision scores, the ML models, particularly the GBDT model, achieved a higher recall rate (> 0.96) without negatively impacting on precision (> 0.98). Similar behavior was observed using the GBDT model in relation to the different mutation types tested, achieving a F1-Score of 0.976, 0.952 and 0.966 for SNVs, insertions and deletions respectively (Additional file 2: Table S1).

**Fig. 4:**
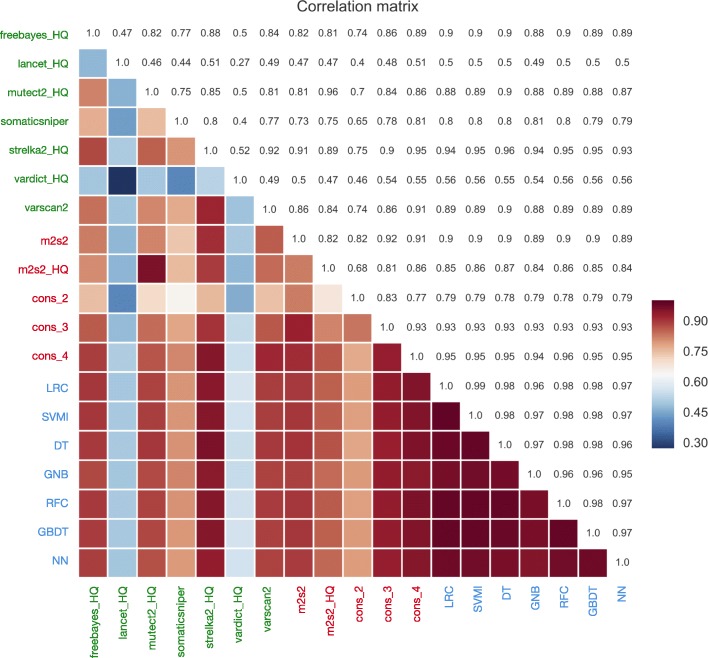
Correlation matrix plot: Pairwise comparison correlation matrix heatmap of some methods results. Half heatmap is represented with colors while the other half is represented with numbers (both halfs represent the same). For those variant callers having FILTER column (“toolname”_HQ), only those variants having “PASS” tag were selected. The methods were divided in three main strategies for better visualization: Machine learning based (blue), standard filtering based (red) and individual tools results (green)

The results between the various approaches were further assessed in a correlation matrix between each pair of methods (Fig. 4). As reported in previous studies [3–11] the individual variant callers had a large degree of disagreement between each other. Consensus strategies have an improved concordance between each other, and the ML methods overall showed a much improved and higher positive correlation (although predictions made by the 7 ML classifiers are from the same training data set, generated from the ensemble results of the seven variant callers). Nevertheless, the ML predictions were not only consistent between each other, but also consistently accurate, with a mean accuracy of 0.9833 (0.0031); mean precision: 0.9813 (0.0111); mean recall 0.9555 (0.0075), mean F1-Score 0.9682 (0.0057), mean FDR: 0.0187 (0.0111), mean MCC: 0.957 (0.0079) and mean AUC: 0.9901 (0.0069).

### Robust performance of ensemble ML based classifiers across mutation types and decreasing variant allelic frequencies

**Fig. 5:**
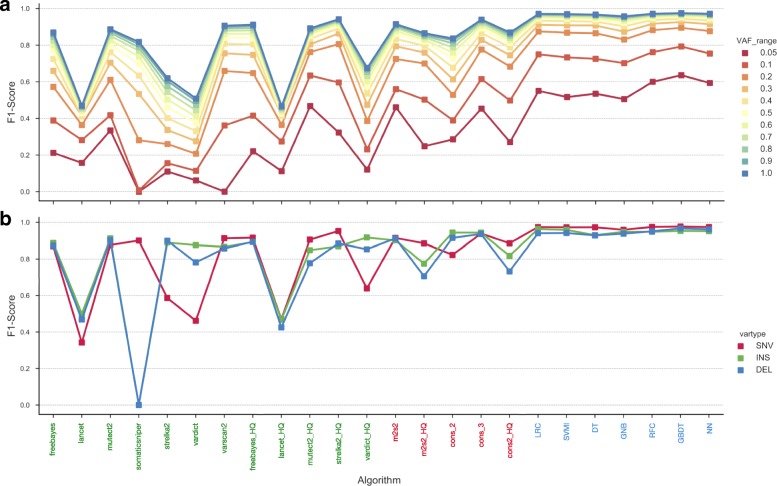
**a**) F1-Scores of each evaluated method for each VAF range. **b**) F1-Scores of each evaluated method according to variant type. The methods were divided in three main strategies in the x-axis: Machine learning based (blue), standard filtering based (red) and individual tools results (green). Each VAF range considered is represented with a different color

The effective interrogation of low-frequency mutations has a great impact in how the performance of variant callers results agree with each other [4, 25, 46]. Thus, the performance of each approach was evaluated across a range of VAFs (Fig. 5a) and different mutation types (SNV, small insertion and deletion) (Fig. 5b). The F1-score was selected as a suitable metric to illustrate both precision and sensitivity simultaneously. The ML models outperform both the individual variant callers and standard filtering protocols across all the VAF scenarios and for all mutation types. The GBDT model offered improved performance overall, but considerably greater performance for those variants thought to be difficult to detect (VAF < 0.05) (Fig. 5a).

**Fig. 6:**
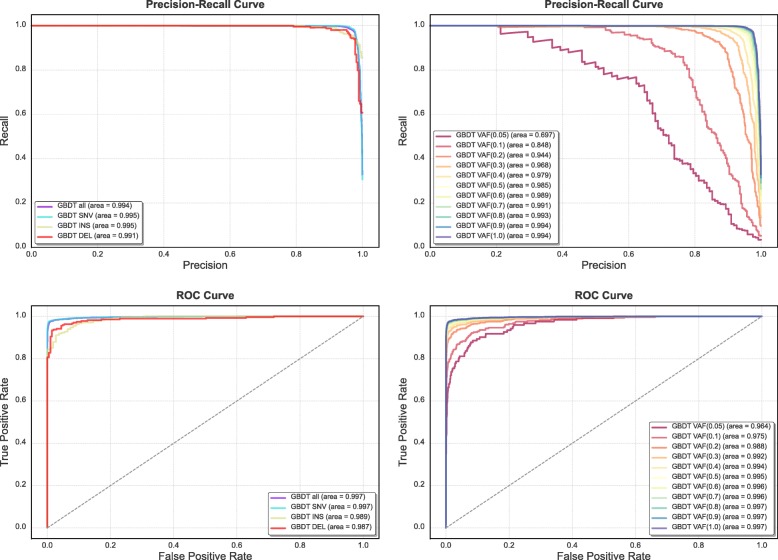
Precision-recall (PR) and Receiver operating characteristic (ROC) curves for GBDT attending to variant type and variant allele frequency (VAF). Correspondent AUC values were computed for all the curves

The results of GBDT were examined in more depth through precision-recall (PR) and standard receiver operating characteristic (ROC) curves, for each allele frequency range, and for each variant type in order to evaluate the classifier under the different conditions (Fig. 6). The PR curves are more informative due to the unbalanced distribution of the data, which comprised of 8918 negative and 3235 positive variants, conferring an over optimistic view in the ROC curves as observed in Fig. 6. As expected, an overall reduction in accuracy at lower VAFs and in indels was observed, due to the additional complexity in the detection of genomic alterations that arise in subclonal tumor cell populations (low-frequency tumor variants), or affecting more than one base pair in the genome.

**Fig. 7:**
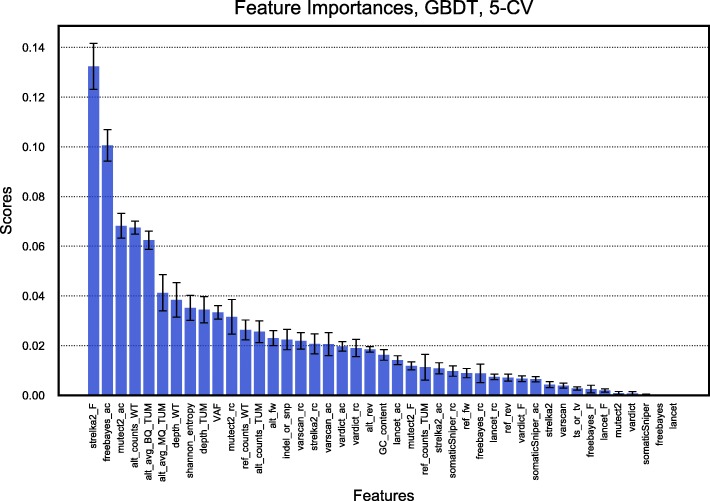
Bar chart of 5-CV GBDT feature importance ranking. The importance of a feature is computed as the (normalized) total reduction of the criterion brought by that feature. The features contributing most to the prediction variable are represented in the left of the plot with highest relative importance scores. The error bar represents the standard deviation across the 5 folds

In addition to VAF, tumor read depth was also evaluated as having a high impact (Additional file 1: Figure S1). Not surprisingly, the vast majority of FP calls originated in low VAF or in those regions with low coverage. Notably, the GBDT classifier was able to discard a substantial number of those FP calls without losing true somatic mutations. The importance of each feature that contributes to the prediction of the correct somatic variant status in the high performing GBDT model was then assessed. Figure 7 shows the computed relative importance of each feature. Once the trees in GBDT were constructed, the importance scores were retrieved for each feature, allowing attributes to be ranked and compared to each other. The Strelka2 filter status (Strelka2_F) contributed most to the learning, followed by tool specific read counts and base and mapping qualities. As can be observed in Fig. 7, the majority of input features provide useful information to the learning model supporting the minimalized but appropriate feature selection process. Moreover, additional experiments excluding different feature sets were carried out for the ML training (Additional file 1: Table S7), and their exclusion had a significant detrimental impact on predictive performance, reinforcing the importance of the selected feature subset.

### Model consistency in independent tests

The S2, S3 and S4 simulations were used as independent tests to evaluate further the performance of the S1 trained ML models. The goal of these independent tests was to highlight model adaptability to different datasets and to evaluate overfitting on the training data. GBDT was trained using the S1 in silico variants and then tested on the S2, S3 and S4 datasets, separately. The performance for GBDT in the independent tests was significantly higher than each of the standard filtering approaches as previously reported by the 5-fold CV experiment (Table 6). GBDT substantially outperformed the best F1-Score score obtained using strict standard filter (the consensus of at least four variant calling tools). Additionally, the concordance of the results was higher in GBDT too, giving more reproducible results across the independent experiments than the other methods, probably due to the model’s ability to infer the interplay between different biological annotations and thus, perform more effective parameter combination (Additional file 2: S2 - S4).

**Table 6:** F1-Scores obtained for the best standard filtering approach and best ML classifier across the different simulations. The consensus call of at least four out of the 7 tools was the winner option for the first category while GBDT model for the second

Method	Category	Description	S1	S2	S3	S4
GBDT	ML	Gradient boosting decision tree	0.9742	0.9762	0.9658	0.8748
cons_4	Standard filtering	Consensus of > = 4 tools	0.9139	0.9326	0.9203	0.8044

## Discussion

Rule-based filtering and consensus voting of multiple variant calling methods can often remove the vast majority of FP calls, unfortunately at the expense missing many TP hits. However, the NeoMutate workflow tries to correct this by taking advantage of ML algorithms that may enhance specificity and methods that reinforce sensitivity, potentially capturing a more complete mutational profile of the tumor with a significant improvement in detection accuracy.

In order to simulate somatic variants in a bona fide cancer context, we used cancer relevant mutations from COSMIC to spike in variants into actual whole-exome sequencing (WES) data from GIAB reference samples. The performance of 7 ML classifiers was comprehensively evaluated under CV and independent tests. We showed that ensemble variant calling boosted with ML, led to a substantially improved performance over standard filtering protocols, in particular decision-tree type models, which displayed a higher sensitivity and specificity balance compared to all of the individual variant callers. Additionally, NeoMutate also outperformed rule-based filtering and simple consensus approaches across a range of variant allele frequencies and mutation types. Performance of GBDT was consistent between the CV on the training data and on the independent test sets, indicating that the model was not subject to overfitting. The GBDT classifier had improved performance overall, but considerably greater performance for those variants thought to be difficult to detect (VAF < 0.05). Several studies have shown the significance of the low frequency variants in terms of their correlation with response to treatment, due to the acquisition of treatment-resistant genetic alterations in the tumor subclones [11, 14, 25]. Thus, the correct detection of low frequency variants in the primary tumor becomes a crucial analysis that will dramatically influence the choice of therapy [11].

Each candidate variant was annotated with non-redundant biological and sequence features. The motivation behind curating the minimal amount of non-redundant features and not all possible features across all possible variant callers is based on the assumption that doing the latter would decrease the portability and scalability of the model. One of the main advantages of using an ensemble of multiple callers boosted by ML as a variant filtering strategy is that the classifiers do not rely on manually defined parameters. The ML models may automatically over time learn these parameters and attempt to capture the interplay between different features. This study incorporated 7 different state-of-the art tools, however, as new variant calling methods are developed, they can be integrated easily due to NeoMutate’s degree of modularity and adaptability. However, the benefit of adding new tools may have the greatest value if the new tool contributes to the detection of de novo true positive predictions. Since the ML framework is built upon an ensemble of embedded tools, incorporating a novel algorithm that can uniquely detect challenging variants could lead to an improved detection accuracy.

## Conclusions

The NeoMutate workflow incorporates a ML framework, where the strengths of multiple callers are exploited using a non-redundant set of biological and sequence features to boost accuracy. We have shown substantially improved performance over standard filtering protocols, specially balancing the trade-off between sensitivity to low-frequency variants and calling too many false positives, critical for an adequate decision making in personalized medicine. Moreover, ML does not rely on user-defined parameters, providing enough flexibility to deal with the common challenges present in clinical tumor samples, such as intra-heterogeneity and normal tissue admixtures. Given the unique and complex characteristics of each tumor, we demonstrate here that integrating multiple tools comprehensively in an ensemble ML layer optimizes somatic variant detection rates, leading to a potential enhancement in cancer diagnosis and treatment response.

## Additional files

Additional file 1: Supplementary materials and methods. Detailed description of all the data generated during the study, including input data overview, in silico spiked-in variants summary, BAMSurgeon performance evaluation, used third-party tools commands. (PDF 1188 kb)

Additional file 2: Comprehensive survey of ensemble ML, consensus filtering strategies and individual variant caller results across the four (S1 – S4) experiments. (XLSX 124 kb)

Additional file 3: Evaluation of individual and combined variant callers on S1 experiment. Exhaustive evaluation of the composite variant callers and all possible unions and intersections between the included algorithms. (PDF 695 kb)

## Abbreviations

GBDT: Gradient boosting decision tree
GIAB: Genome in a Bottle SNV Single nucleotide variant VAF Variant allele frequency ML Machine learning
NGS: Next generation sequencing
PR: Precision-recall
ROC: Receiver operating characteristic
WES: Whole exome sequencing
