# Supplementary material for: NeoMutate: an ensemble machine learning framework for the prediction of somatic mutations in cancer
Source: BMC Med Genomics. 2019 May 16;12:63. doi: 10.1186/s12920-019-0508-5 (PMC6524241; doi:10.1186/s12920-019-0508-5)
Supplement: Supplementary file 1 — Supplementary materials and methods. Detailed description of all the data generated during the study, including input data overview, in silico spiked-in variants summary, BAMSurgeon performance evaluation, used third-party tools commands. (PDF 1188 kb) [file 12920_2019_508_MOESM1_ESM.pdf]

Metric	Description
**sequences**	Number of sequences (reads)
**1st fragments**	Number of reads in first pair
**last fragments**	Number of reads in second pair
**reads mapped**	Number of mapped reads
**reads mapped and paired**	Paired-end technology bit set and both mates mapped
**reads unmapped**	Number of reads unmapped
**reads properly paired**	Number of reads properly paired (proper-pair bit set)
**reads paired**	Paired-end technology bit set
**reads duplicated**	Number of reads duplicated
**reads MQ0**	Number of reads with mapping quality 0
**non-primary alignments**	Number of non-primary alignments
**bases mapped**	Number of bases mapped by at least one read (ignores clipping)
**bases mapped (cigar)**	Number of bases mapped according to cigar string (more accurate)
**bases trimmed**	Number of bases
**mismatches**	Mismatches extracted from NM field.
**error rate**	Mismatches/basses mapped (cigar)
**average length**	Average length mapped reads
**maximum length**	Maximum length mapped reads
**average quality**	Average mapping quality
**insert size average**	Insert size mean
**insert size standard deviation**	Insert size std
**inward oriented pairs**	--> <--
**outward oriented pairs**	<-- -->
**pairs with other orientation**	Other orientations different from (--> <-- and <-- -->)
**pairs on different chromosomes**	Read pairs mapping in different chromosomes

Table S1. **Mapping quality evaluation report**. Description of the collected survey of metrics during mapping quality check performed by NeoMutate.

Sample ID	Mapped reads	Split ratio (T|N)	Tumor_reads	Normal_reads
**NA12878**	164883031	0.5|0.5	82427580	82403048
**NA12891**	163350232	0.5|0.5	81621882	81578248
**NA24631**	114979364	0.6|0.4	68902286	45933652

Table S2. **BAM split ratio**. Split ratio applied to the initial BAM file in order to generate the paired tumor-normal BAM subsets. 0.5 indicates a perfect division into two equal sized sub-BAM files. 0.6|0.4 ratio generates a tumor BAM file containing the 60% of the initial reads and a normal BAM file with the remaining 40%. BAM split process was conducted using bamsplit_proportion.py tool available in BAMSurgeon repository.

Simulation ID	Sample ID	Number	VAF_mean	VAF_stdev	VAF_median	VAF_min	VAF_max
**All variants**							
**S1**	NA12878	3600	0.5140	0.2906	0.5160	0.0100	1.0000
**S2**	NA12891	6000	0.5138	0.2904	0.5080	0.0100	1.0000
**S3**	NA24631	6000	0.5153	0.2911	0.5260	0.0100	1.0000
**S4**	NA24631	5000	0.1053	0.0430	0.1055	0.0117	0.1984
**SNVs**							
**S1**	NA12878	3000	0.5170	0.2889	0.5230	0.0100	1.0000
**S2**	NA12891	5000	0.5130	0.2903	0.5080	0.0100	1.0000
**S3**	NA24631	4000	0.5122	0.2917	0.5180	0.0100	1.0000
**S4**	NA24631	3000	0.1049	0.0426	0.1060	0.0117	0.1984
**Insertions**							
**S1**	NA12878	300	0.5017	0.2925	0.4960	0.0150	1.0000
**S2**	NA12891	500	0.5170	0.2910	0.5140	0.0100	1.0000
**S3**	NA24631	1000	0.5129	0.2925	0.5250	0.0120	1.0000
**S4**	NA24631	1012	0.1057	0.0440	0.1048	0.0136	0.1958
**Deletions**							
**S1**	NA12878	300	0.4976	0.3060	0.4790	0.0130	1.0000
**S2**	NA12891	500	0.5181	0.2927	0.5080	0.0150	1.0000
**S3**	NA24631	1000	0.5303	0.2878	0.5450	0.0130	1.0000
**S4**	NA24631	988	0.1060	0.0435	0.1042	0.0143	0.1973

Table S3. VAF range of the synthetic variants simulated at each experiment.

All			
**Simulation ID**	**Selected**	**Added**	**%**
**S1**	3600	3421	95.03
**S2**	6000	5573	92.88
**S3**	6000	5763	96.05
**S4**	5000	4894	97.88
**SNVs**			
**Simulation ID**	**Selected**	**Added**	**%**
**S1**	3000	2829	94.30
**S2**	5000	4646	92.92
**S3**	4000	3897	97.43
**S4**	3000	2900	96.67
**Insertion**			
**Simulation ID**	**Selected**	**Added**	**%**
**S1**	300	297	99.00
**S2**	500	437	87.40
**S3**	1000	892	89.20
**S4**	1012	1009	99.70
**Deletion**			
**Simulation ID**	**Selected**	**Added**	**%**
**S1**	300	295	98.33
**S2**	500	490	98.00
**S3**	1000	974	97.40
**S4**	988	985	99.70

Table S4. **BAMSurgeon performance evaluation.** As can be observed not all the input selected variants were successfully added to the BAM files probably due to low depth or extremely low VAF issues.

<table>
<tr><th>Tool</th><th>Command</th></tr>
<tr><td>MuTect2</td><td>GATK -T MuTect2 -R hs37d5.fa -I:tumor normal.bam -L exomic_regions.bed --artifact_detection_mode -o PON.vcf
GATK -T MuTect2 -R hs37d5.fa -I:tumor tumor.bam -I:normal normal.bam -PON PON.vcf --dbsnp 00-All.vcf.gz --cosmic v83_GRCh37_cosmic.vcf.gz -L exomic_regions.bed -o mutect2_output.vcf</td></tr>
<tr><td>VarDict</td><td>VarDict -G hs37d5.fa -f 0.01 -N tum -b "tumor.bam|normal.bam" -C -c 1 -S 2 -E 3 -g 4 exomic_regions.bed | testsomatic.R | var2vcf_paired.pl -P 0.9 -N "tumor|normal" -f 0.01 > vardict_output.vcf
bam-somaticsniper -F vcf -Q 15 -q 10 -J -s 0.0010 -G -L -f hs37d5.fa tumor.bam normal.bam somaticsniper_output.vcf</td></tr>
<tr><td>Strelka2</td><td>strelka2 --tumorBam=.tumor.bam --normalBam=normal.bam --referenceFasta=hs37d5.fa --exome --callRegions=exomic_regions.bed.gz --runDir=strelka2/strelka2_output</td></tr>
<tr><td>VarScan2</td><td>samtools mpileup -l exomic_regions.bed -B -f hs37d5.fa normal.bam tumor.bam | varscan2 somatic --mpileup varscan_output --output-vcf 1 --min-coverage-normal 3 --min-coverage-tumor 3 --p-value 0.10 --strand-filter 1 --min-var-freq 0.08 --min-coverage 3 --normal-purity 1.0 --tumor-purity 0.9
varscan2 processSomatic varscan_output.join.vcf
awk 'BEGIN {OFS="\t"} {if (!/^#/) { isDel=(length($4) > length($5)) ? 1 : 0; print $1,($2+isDel),($2+isDel); }}' varscan_output.join.Somatic.hc.vcf > varscan_output.join.Somatic.hc.var
bam-readcount -q 1 -b 20 -w 0 -l varscan_output.join.Somatic.hc.var -f hs37d5.fa tumor.bam > varscan_output.join.Somatic.hc.rc
varscan2 fpfilter varscan_output.join.Somatic.hc.vcf tum_varscan.join.Somatic.hc.rc --output-file varscan_output.join.Somatic.hc.fpfilter.vcf --filtered-file varscan_output.join.Somatic.hc.fpfilter.FAIL.vcf --min-ref-avgrl 35 --min-var-avgrl 35</td></tr>
<tr><td>Freebayes</td><td>speedseq somatic -o freebayes_output -C 2 -F 0.02 hs37d5.fa normal.bam tumor.bam</td></tr>
<tr><td>SomaticSniper</td><td>bam-somaticsniper -F vcf -Q 15 -q 10 -J -s 0.0010 -G -L -f hs37d5.fa tumor.bam normal.bam somaticsniper_output.vcf</td></tr>
<tr><td>Lancet</td><td>lancet --tumor tumor.bam --normal normal_bam --ref hs37d5.fa --bed exomic_regions.bed</td></tr>
</table>

Table S5. **Commands used for running each individual algorithm included in the ensemble of variant callers.**

Strategy	Description
**Freebayes_ss_HQ**	Variants reported by freebayes having PASS in the FILTER column (high-confidence variants)
**Lancet_HQ**	Variants reported by lancet having PASS in the FILTER column (high-confidence variants)
**MuTect2_HQ**	Variants reported by MuTect2 having PASS in the FILTER column (high-confidence variants)
**Strelka2_HQ**	Variants reported by Strelka2 having PASS in the FILTER column (high-confidence variants)
**VarDict_HQ**	Variants reported by VarDict having PASS in the FILTER column (high-confidence variants)
**m2s2**	Intersection of mutect2 and strelka2
**m2s2_HQ**	Intersection PASS variants reported by mutect2 and strelka2
**cons_2**	Keep variants reported by >= 2 tools
**cons_3**	Keep variants reported by >= 3 tools
**cons_4**	Keep variants reported by >= 4 tools
**cons_5**	Keep variants reported by >= 5 tools
**cons_6**	Keep variants reported by >= 6 tools
**cons_7**	Keep variants reported by >= 7 tools
**cons_2_HQ**	Keep variants reported by >= 2 tools tagged as PASS

Table S6. Some of the commonly used variant calling filtering strategies. The results of machine learning classifiers were compared against the described standard filtering protocols in addition to individual tools raw results.

Model	Description	TP	FP	FN	TN	accuracy	precision	recall	F1-Score	FDR	AUC
**LR**	Logistic regression	3071	45	164	8873	0.9828	0.9856	0.9493	0.9671	0.0144	0.9894
**SVMl**	Support vector machine	3067	54	168	8864	0.9817	0.9827	0.9481	0.9651	0.0173	0.9707
**DT**	Decision tree	3054	39	181	8879	0.9819	0.9874	0.9441	0.9652	0.0126	0.9810
**GNB**	Gaussian Naïve Bayes	3093	161	142	8757	0.9751	0.9505	0.9561	0.9533	0.0495	0.9809
**RF**	Random forest	3075	65	160	8853	0.9815	0.9793	0.9505	0.9647	0.0207	0.9843
**GBDT**	Gradient boosting decision tree	3078	58	157	8860	0.9823	0.9815	0.9515	0.9663	0.0185	0.9910
**NN**	Neural network	3072	74	163	8844	0.9805	0.9765	0.9496	0.9629	0.0235	0.9828

Table S7. S1 experiment results with BAM level extracted features excluded.

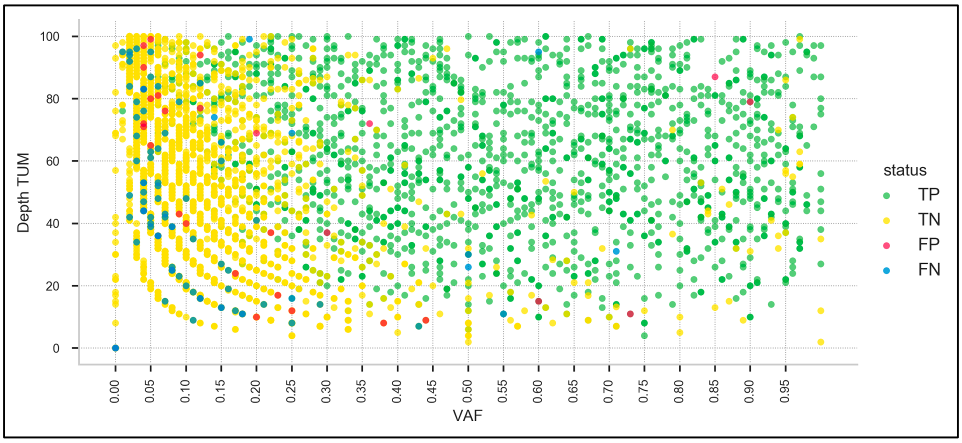

Fig. S1: GBDT predicted variants with correspondent call status as a function of VAF and number of reads in the tumor sample. Decreasing VAF and tumor depth makes the differentiation of true variant from background noise tricky, originating false positive calls to be reported by the one or more tools. However, trained GBDT showed an improved ability to detect them and classify them as true-negative events.
