# Supplementary material for: NeoMutate: an ensemble machine learning framework for the prediction of somatic mutations in cancer
Source: BMC Med Genomics. 2019 May 16;12:63. doi: 10.1186/s12920-019-0508-5 (PMC6524241; doi:10.1186/s12920-019-0508-5)
Supplement: Supplementary file 3 — Evaluation of individual and combined variant callers on S1 experiment. Exhaustive evaluation of the composite variant callers and all possible unions and intersections between the included algorithms. (PDF 695 kb) [file 12920_2019_508_MOESM3_ESM.pdf]

Evaluation of individual and combined variant callers

The performance of the 7 variant calling algorithms was checked as forming the main input for the machine learning framework. As mentioned, the complexity of the input data in addition to the dissimilar mathematical models applied by the different callers resulted in divergent variant set prediction for each tool (Fig. S1a). The figure shows the raw calls reported by each tool. Considering only those variants reported as high confidence variants (those having "PASS" tag in the corresponding VCF "FILTER" column), Strelka2 reported 2907 (98.74%) TP and 37 (1.26%) FP, and MuTect2 2620 (99.13%) TP and 23 (0.87%) FP, becoming the 2 best individual tools for S1 simulation. The concordance of the calls among the tools was checked (Fig. S1b). The assumption is that the more tools predict a given variant, the more confident and true-likely it is. However, as have been mentioned, due to tool uniqueness and specificity to predict a given type of variant, some relevant mutations may only be predicted by a subset of the tools (as the 59 TP variants detected by a single tool in S1 simulation). Not only that but some true variants were not reported by any caller for various reasons, being low allele frequency the most remarkable factor. 162 (5%), 228 (4.1%), 218 (3.8%) and 1102 (22.52%) true variants were not detected by any of the seven variant callers for S1, S2, S3 and S4 respectively. Fig. S1b highlights the suitability of the intersection of tools strategy when precision is the main goal of the study. However, the sensitivity (recall) is dramatically harmed, missing the correct identification of challenging variants in cancer, as those present in a subset of tumor cells (i.e. subclonal mutations). See Table S1 for a more detailed overview of all possible combinations (intersections and unions) of the different independent tools.

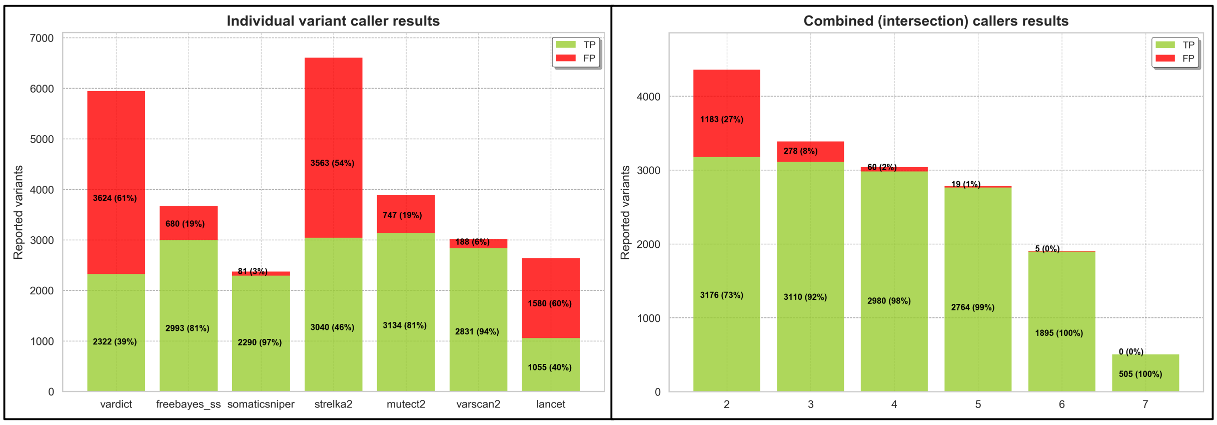

Fig. S1: a) Raw somatic variant candidates (not filtered) reported by each tool in S1 experiment. As can be observed some tools show a more conservative behaviour, while others seem to be more daring due to the underlying mathematical models. **b) Concordance of calls among somatic variant callers for S1 experiment**. Each bar represents the number of calls shared by at least the corresponding number of tools.

Tool combination	Approach	Ground truth	TP	FP	FN	precision	recall	F1-Score
vardict freebayes ss strelka2 mutect2 varscan2 lancet	union	3397	3235	8899	0	0.26661	1	0.42098
vardict freebayes ss somaticsniper strelka2 mutect2 varscan2 lancet	union	3397	3235	8918	0	0.26619	1	0.42046
vardict freebayes ss strelka2 mutect2 varscan2	union	3397	3233	7464	2	0.30223	0.99938	0.46411
vardict freebayes ss somaticsniper strelka2 mutect2 varscan2	union	3397	3233	7483	2	0.3017	0.99938	0.46348
vardict freebayes ss somaticsniper strelka2 varscan2 lancet	union	3397	3232	8661	3	0.27176	0.99907	0.42729
vardict freebayes ss somaticsniper strelka2 mutect2 lancet	union	3397	3232	8853	3	0.26744	0.99907	0.42193
vardict freebayes ss strelka2 varscan2 lancet	union	3397	3231	8642	4	0.27213	0.99876	0.42772
vardict somaticsniper strelka2 mutect2 varscan2 lancet	union	3397	3231	8685	4	0.27115	0.99876	0.42651
vardict freebayes ss strelka2 mutect2 lancet	union	3397	3231	8834	4	0.2678	0.99876	0.42235
vardict freebayes ss somaticsniper strelka2 varscan2	union	3397	3230	7224	5	0.30897	0.99845	0.47191
vardict freebayes ss somaticsniper strelka2 mutect2	union	3397	3230	7416	5	0.3034	0.99845	0.46538
vardict freebayes ss strelka2 varscan2	union	3397	3229	7204	6	0.3095	0.99815	0.47249
vardict somaticsniper strelka2 mutect2 varscan2	union	3397	3229	7247	6	0.30823	0.99815	0.47101
vardict freebayes ss strelka2 mutect2	union	3397	3229	7397	6	0.30388	0.99815	0.46591
vardict freebayes ss somaticsniper strelka2 lancet	union	3397	3229	8596	6	0.27307	0.99815	0.42882
vardict strelka2 mutect2 varscan2 lancet	union	3397	3229	8665	6	0.27148	0.99815	0.42686
vardict somaticsniper strelka2 mutect2 lancet	union	3397	3228	8617	7	0.27252	0.99784	0.42812
vardict strelka2 mutect2 varscan2	union	3397	3227	7227	8	0.30869	0.99753	0.47147
vardict freebayes ss strelka2 lancet	union	3397	3227	8577	8	0.27338	0.99753	0.42915
vardict freebayes ss somaticsniper strelka2	union	3397	3226	7157	9	0.3107	0.99722	0.47378
vardict somaticsniper strelka2 mutect2	union	3397	3226	7177	9	0.3101	0.99722	0.47309
vardict strelka2 mutect2 lancet	union	3397	3225	8596	10	0.27282	0.99691	0.4284
vardict freebayes ss strelka2	union	3397	3224	7137	11	0.31117	0.9966	0.47426
vardict strelka2 mutect2	union	3397	3223	7156	12	0.31053	0.99629	0.47348
vardict somaticsniper strelka2 varscan2 lancet	union	3397	3223	8398	12	0.27734	0.99629	0.4339
vardict freebayes ss mutect2 varscan2 lancet	union	3397	3221	6185	14	0.34244	0.99567	0.50961
vardict freebayes ss somaticsniper mutect2 varscan2 lancet	union	3397	3221	6211	14	0.3415	0.99567	0.50857
vardict somaticsniper strelka2 varscan2	union	3397	3221	6958	14	0.31644	0.99567	0.48024
vardict somaticsniper strelka2 lancet	union	3397	3220	8330	15	0.27879	0.99536	0.43558
vardict freebayes ss mutect2 varscan2	union	3397	3218	4728	17	0.40498	0.99474	0.57562

vardict freebayes ss somaticsniper mutect2 varscan2	union	3397	3218	4754	17	0.40366	0.99474	0.57428
vardict somaticsniper strelka2	union	3397	3217	6888	18	0.31836	0.99444	0.48231
vardict somaticsniper mutect2 varscan2 lancet	union	3397	3216	5728	19	0.35957	0.99413	0.52812
vardict freebayes ss somaticsniper mutect2 lancet	union	3397	3216	6120	19	0.34447	0.99413	0.51165
vardict freebayes ss mutect2 lancet	union	3397	3215	6093	20	0.3454	0.99382	0.51264
vardict somaticsniper mutect2 varscan2	union	3397	3213	4268	22	0.42949	0.9932	0.59966
vardict mutect2 varscan2 lancet	union	3397	3213	5676	22	0.36146	0.9932	0.53002
vardict freebayes ss somaticsniper mutect2	union	3397	3212	4661	23	0.40798	0.99289	0.57832
vardict freebayes ss mutect2	union	3397	3211	4634	24	0.40931	0.99258	0.5796
vardict mutect2 varscan2	union	3397	3210	4216	25	0.43227	0.99227	0.60219
vardict somaticsniper mutect2 lancet	union	3397	3210	5629	25	0.36316	0.99227	0.53172
vardict somaticsniper mutect2	union	3397	3206	4167	29	0.43483	0.99104	0.60445
vardict freebayes ss somaticsniper varscan2 lancet	union	3397	3206	5805	29	0.35579	0.99104	0.5236
vardict freebayes ss varscan2 lancet	union	3397	3205	5779	30	0.35675	0.99073	0.52459
vardict mutect2 lancet	union	3397	3204	5574	31	0.365	0.99042	0.53342
freebayes ss strelka2 mutect2 varscan2 lancet	union	3397	3202	5878	33	0.35264	0.9898	0.52002
freebayes ss somaticsniper strelka2 mutect2 varscan2 lancet	union	3397	3202	5899	33	0.35183	0.9898	0.51913
vardict strelka2 varscan2 lancet	union	3397	3202	8378	33	0.27651	0.9898	0.43226
vardict mutect2	union	3397	3200	4112	35	0.43764	0.98918	0.60681
vardict freebayes ss somaticsniper varscan2	union	3397	3200	4338	35	0.42452	0.98918	0.59408
vardict freebayes ss varscan2	union	3397	3199	4311	36	0.42597	0.98887	0.59544
vardict strelka2 varscan2	union	3397	3199	6937	36	0.31561	0.98887	0.4785
freebayes ss somaticsniper strelka2 mutect2 lancet	union	3397	3198	5791	37	0.35577	0.98856	0.52323
vardict strelka2 lancet	union	3397	3198	8309	37	0.27792	0.98856	0.43386
freebayes ss strelka2 mutect2 lancet	union	3397	3197	5770	38	0.35653	0.98825	0.52401
freebayes ss strelka2 mutect2 varscan2	union	3397	3196	4372	39	0.4223	0.98794	0.59169
freebayes ss somaticsniper strelka2 mutect2 varscan2	union	3397	3196	4393	39	0.42114	0.98794	0.59054
vardict strelka2	union	3397	3194	6866	41	0.3175	0.98733	0.48048
freebayes ss somaticsniper strelka2 mutect2	union	3397	3192	4283	43	0.42702	0.98671	0.59608
freebayes ss strelka2 mutect2	union	3397	3191	4262	44	0.42815	0.9864	0.59712
vardict freebayes ss somaticsniper lancet	union	3397	3189	5711	46	0.35831	0.98578	0.52559
somaticsniper strelka2 mutect2 varscan2 lancet	union	3397	3186	5629	49	0.36143	0.98485	0.5288
strelka2 mutect2 varscan2 lancet	union	3397	3183	5604	52	0.36224	0.98393	0.52953
freebayes ss mutect2 varscan2 lancet	union	3397	3182	3018	53	0.51323	0.98362	0.67451

freebayes ss somaticsniper mutect2 varscan2 lancet	union	3397	3182	3046	53	0.51092	0.98362	0.67251
vardict freebayes ss lancet	union	3397	3182	5684	53	0.3589	0.98362	0.52591
somaticsniper strelka2 mutect2 lancet	union	3397	3181	5514	54	0.36584	0.98331	0.53328
freebayes ss somaticsniper strelka2 varscan2 lancet	union	3397	3181	5531	54	0.36513	0.98331	0.53252
somaticsniper strelka2 mutect2 varscan2	union	3397	3179	4118	56	0.43566	0.98269	0.60368
freebayes ss strelka2 varscan2 lancet	union	3397	3179	5510	56	0.36586	0.98269	0.53321
vardict freebayes ss somaticsniper	union	3397	3177	4242	58	0.42822	0.98207	0.5964
strelka2 mutect2 lancet	union	3397	3177	5488	58	0.36665	0.98207	0.53395
freebayes ss somaticsniper mutect2 lancet	union	3397	3176	2909	59	0.52194	0.98176	0.68155
strelka2 mutect2 varscan2	union	3397	3176	4093	59	0.43692	0.98176	0.60472
vardict somaticsniper varscan2 lancet	union	3397	3176	5264	59	0.3763	0.98176	0.54407
freebayes ss somaticsniper strelka2 lancet	union	3397	3176	5420	59	0.36947	0.98176	0.53689
freebayes ss mutect2 lancet	union	3397	3174	2880	61	0.52428	0.98114	0.68339
somaticsniper strelka2 mutect2	union	3397	3174	4001	61	0.44237	0.98114	0.6098
freebayes ss mutect2 varscan2	union	3397	3173	1474	62	0.68281	0.98083	0.80513
freebayes ss somaticsniper mutect2 varscan2	union	3397	3173	1502	62	0.67872	0.98083	0.80228
freebayes ss strelka2 lancet	union	3397	3173	5399	62	0.37016	0.98083	0.53748
vardict somaticsniper varscan2	union	3397	3170	3794	65	0.4552	0.97991	0.62163
strelka2 mutect2	union	3397	3170	3975	65	0.44367	0.97991	0.61079
vardict freebayes ss	union	3397	3169	4214	66	0.42923	0.9796	0.59691
freebayes ss somaticsniper strelka2 varscan2	union	3397	3168	4017	67	0.44092	0.97929	0.60806
freebayes ss somaticsniper mutect2	union	3397	3166	1363	69	0.69905	0.97867	0.81556
freebayes ss strelka2 varscan2	union	3397	3166	3995	69	0.44212	0.97867	0.60908
somaticsniper mutect2 varscan2 lancet	union	3397	3165	2512	70	0.55751	0.97836	0.71028
freebayes ss mutect2	union	3397	3164	1334	71	0.70342	0.97805	0.81831
freebayes ss somaticsniper strelka2	union	3397	3162	3904	73	0.4475	0.97743	0.61392
mutect2 varscan2 lancet	union	3397	3161	2453	74	0.56306	0.97713	0.71443
freebayes ss strelka2	union	3397	3159	3882	76	0.44866	0.97651	0.61483
somaticsniper mutect2 varscan2	union	3397	3155	963	80	0.76615	0.97527	0.85815
somaticsniper mutect2 lancet	union	3397	3154	2360	81	0.572	0.97496	0.721
mutect2 varscan2	union	3397	3151	904	84	0.77707	0.97403	0.86447
mutect2 lancet	union	3397	3145	2298	90	0.57781	0.97218	0.72482
vardict varscan2 lancet	union	3397	3144	5207	91	0.37648	0.97187	0.54272
somaticsniper mutect2	union	3397	3143	809	92	0.79529	0.97156	0.87463
somaticsniper strelka2 varscan2 lancet	union	3397	3139	5229	96	0.37512	0.97032	0.54107
vardict varscan2	union	3397	3135	3736	100	0.45627	0.96909	0.62042
somaticsniper strelka2 lancet	union	3397	3133	5111	102	0.38003	0.96847	0.54587

somaticsniper strelka2 varscan2	union	3397	3122	3710	113	0.45697	0.96507	0.62024
somaticsniper strelka2	union	3397	3115	3590	120	0.46458	0.96291	0.62676
freebayes ss somaticsniper varscan2 lancet	union	3397	3111	2436	124	0.56084	0.96167	0.70849
freebayes ss varscan2 lancet	union	3397	3109	2408	126	0.56353	0.96105	0.71047
vardict somaticsniper lancet	union	3397	3100	5160	135	0.3753	0.95827	0.53936
freebayes ss somaticsniper varscan2	union	3397	3073	867	162	0.77995	0.94992	0.85659
strelka2 varscan2 lancet	union	3397	3073	5204	162	0.37127	0.94992	0.53388
freebayes ss somaticsniper lancet	union	3397	3072	2282	163	0.57378	0.94961	0.71533
freebayes ss varscan2	union	3397	3071	838	164	0.78562	0.9493	0.85974
strelka2 lancet	union	3397	3066	5085	169	0.37615	0.94776	0.53856
vardict somaticsniper	union	3397	3064	3688	171	0.45379	0.94714	0.6136
freebayes ss lancet	union	3397	3054	2253	181	0.57547	0.94405	0.71506
strelka2 varscan2	union	3397	3048	3684	187	0.45276	0.94219	0.61162
freebayes ss somaticsniper	union	3397	3015	710	220	0.8094	0.93199	0.86638
strelka2 mutect2	intersection	3397	3004	335	231	0.89967	0.92859	0.9139
somaticsniper varscan2 lancet	union	3397	3001	1834	234	0.62068	0.92767	0.74374
freebayes ss mutect2	intersection	3397	2963	93	272	0.96957	0.91592	0.94198
somaticsniper varscan2	union	3397	2937	259	298	0.91896	0.90788	0.91339
varscan2 lancet	union	3397	2910	1765	325	0.62246	0.89954	0.73578
freebayes ss strelka2	intersection	3397	2874	361	361	0.88841	0.88841	0.88841
freebayes ss strelka2 mutect2	intersection	3397	2865	61	370	0.97915	0.88563	0.93004
strelka2 varscan2	intersection	3397	2823	67	412	0.97682	0.87264	0.9218
mutect2 varscan2	intersection	3397	2814	31	421	0.9891	0.86986	0.92566
strelka2 mutect2 varscan2	intersection	3397	2812	28	423	0.99014	0.86924	0.92576
freebayes ss varscan2	intersection	3397	2753	30	482	0.98922	0.851	0.91492
freebayes ss strelka2 varscan2	intersection	3397	2752	22	483	0.99207	0.8507	0.91596
freebayes ss mutect2 varscan2	intersection	3397	2745	13	490	0.99529	0.84853	0.91607
freebayes ss strelka2 mutect2 varscan2	intersection	3397	2745	13	490	0.99529	0.84853	0.91607
vardict lancet	union	3397	2624	5097	611	0.33985	0.81113	0.47901
somaticsniper lancet	union	3397	2559	1659	676	0.60669	0.79104	0.6867
somaticsniper mutect2	intersection	3397	2281	19	954	0.99174	0.7051	0.82421
freebayes ss somaticsniper	intersection	3397	2268	51	967	0.97801	0.70108	0.81671
freebayes ss somaticsniper mutect2	intersection	3397	2261	18	974	0.9921	0.69892	0.82009
vardict mutect2	intersection	3397	2256	259	979	0.89702	0.69737	0.7847
somaticsniper strelka2	intersection	3397	2215	54	1020	0.9762	0.6847	0.80487
somaticsniper strelka2 mutect2	intersection	3397	2210	18	1025	0.99192	0.68315	0.80908
freebayes ss somaticsniper strelka2	intersection	3397	2196	46	1039	0.97948	0.67883	0.8019
freebayes ss somaticsniper strelka2 mutect2	intersection	3397	2192	18	1043	0.99186	0.67759	0.80514
somaticsniper varscan2	intersection	3397	2184	10	1051	0.99544	0.67512	0.80457
somaticsniper strelka2 varscan2	intersection	3397	2183	9	1052	0.99589	0.67481	0.8045
somaticsniper mutect2 varscan2	intersection	3397	2179	7	1056	0.9968	0.67357	0.80391

somaticsniper strelka2 mutect2 varscan2	intersection	3397	2179	7	1056	0.9968	0.67357	0.80391
vardict strelka2	intersection	3397	2168	321	1067	0.87103	0.67017	0.75751
freebayes ss somaticsniper strelka2 varscan2	intersection	3397	2164	8	1071	0.99632	0.66893	0.80044
freebayes ss somaticsniper varscan2	intersection	3397	2164	9	1071	0.99586	0.66893	0.8003
freebayes ss somaticsniper mutect2 varscan2	intersection	3397	2161	7	1074	0.99677	0.66801	0.79993
freebayes ss somaticsniper strelka2 mutect2 varscan2	intersection	3397	2161	7	1074	0.99677	0.66801	0.79993
vardict strelka2 mutect2	intersection	3397	2155	137	1080	0.94023	0.66615	0.77981
vardict freebayes ss	intersection	3397	2146	90	1089	0.95975	0.66337	0.7845
vardict freebayes ss mutect2	intersection	3397	2127	25	1108	0.98838	0.6575	0.78968
vardict freebayes ss strelka2	intersection	3397	2057	42	1178	0.97999	0.63586	0.77128
vardict freebayes ss strelka2 mutect2	intersection	3397	2053	23	1182	0.98892	0.63462	0.77311
vardict varscan2	intersection	3397	2018	76	1217	0.96371	0.6238	0.75737
vardict strelka2 varscan2	intersection	3397	2015	26	1220	0.98726	0.62287	0.76384
vardict mutect2 varscan2	intersection	3397	2011	23	1224	0.98869	0.62164	0.76333
vardict strelka2 mutect2 varscan2	intersection	3397	2010	20	1225	0.99015	0.62133	0.76353
vardict freebayes ss varscan2	intersection	3397	1970	15	1265	0.99244	0.60896	0.75479
vardict freebayes ss strelka2 varscan2	intersection	3397	1969	11	1266	0.99444	0.60866	0.75513
vardict freebayes ss mutect2 varscan2	intersection	3397	1965	8	1270	0.99595	0.60742	0.75461
vardict freebayes ss strelka2 mutect2 varscan2	intersection	3397	1965	8	1270	0.99595	0.60742	0.75461
vardict somaticsniper	intersection	3397	1548	17	1687	0.98914	0.47852	0.645
vardict somaticsniper mutect2	intersection	3397	1545	10	1690	0.99357	0.47759	0.64509
vardict freebayes ss somaticsniper	intersection	3397	1534	15	1701	0.99032	0.47419	0.6413
vardict freebayes ss somaticsniper mutect2	intersection	3397	1532	10	1703	0.99351	0.47357	0.64141
vardict somaticsniper strelka2	intersection	3397	1496	12	1739	0.99204	0.46244	0.63082
vardict somaticsniper strelka2 mutect2	intersection	3397	1494	10	1741	0.99335	0.46182	0.63051
vardict freebayes ss somaticsniper strelka2	intersection	3397	1483	12	1752	0.99197	0.45842	0.62706
vardict freebayes ss somaticsniper strelka2 mutect2	intersection	3397	1482	10	1753	0.9933	0.45811	0.62704
vardict somaticsniper varscan2	intersection	3397	1477	4	1758	0.9973	0.45657	0.62638
vardict somaticsniper strelka2 varscan2	intersection	3397	1477	4	1758	0.9973	0.45657	0.62638
vardict somaticsniper mutect2 varscan2	intersection	3397	1475	4	1760	0.9973	0.45595	0.6258
vardict somaticsniper strelka2 mutect2 varscan2	intersection	3397	1475	4	1760	0.9973	0.45595	0.6258
vardict freebayes ss somaticsniper varscan2	intersection	3397	1464	4	1771	0.99728	0.45255	0.62258
vardict freebayes ss somaticsniper strelka2 varscan2	intersection	3397	1464	4	1771	0.99728	0.45255	0.62258
vardict freebayes ss somaticsniper mutect2 varscan2	intersection	3397	1463	4	1772	0.99727	0.45224	0.62229

vardict freebayes ss somaticsniper strelka2 mutect2 varscan2	intersection	3397	1463	4	1772	0.99727	0.45224	0.62229
mutect2 lancet	intersection	3397	1044	29	2191	0.97297	0.32272	0.48468
strelka2 lancet	intersection	3397	1029	58	2206	0.94664	0.31808	0.47617
strelka2 mutect2 lancet	intersection	3397	1025	20	2210	0.98086	0.31685	0.47897
freebayes ss lancet	intersection	3397	994	7	2241	0.99301	0.30726	0.46931
freebayes ss mutect2 lancet	intersection	3397	993	2	2242	0.99799	0.30696	0.4695
freebayes ss strelka2 lancet	intersection	3397	982	2	2253	0.99797	0.30355	0.46551
freebayes ss strelka2 mutect2 lancet	intersection	3397	982	2	2253	0.99797	0.30355	0.46551
varscan2 lancet	intersection	3397	976	3	2259	0.99694	0.3017	0.46322
strelka2 varscan2 lancet	intersection	3397	975	1	2260	0.99898	0.30139	0.46307
mutect2 varscan2 lancet	intersection	3397	975	1	2260	0.99898	0.30139	0.46307
strelka2 mutect2 varscan2 lancet	intersection	3397	974	1	2261	0.99897	0.30108	0.46271
freebayes ss varscan2 lancet	intersection	3397	953	0	2282	1	0.29459	0.45511
freebayes ss strelka2 varscan2 lancet	intersection	3397	953	0	2282	1	0.29459	0.45511
freebayes ss mutect2 varscan2 lancet	intersection	3397	953	0	2282	1	0.29459	0.45511
freebayes ss strelka2 mutect2 varscan2 lancet	intersection	3397	953	0	2282	1	0.29459	0.45511
somaticsniper lancet	intersection	3397	786	2	2449	0.99746	0.24297	0.39075
somaticsniper mutect2 lancet	intersection	3397	786	2	2449	0.99746	0.24297	0.39075
freebayes ss somaticsniper lancet	intersection	3397	782	1	2453	0.99872	0.24173	0.38925
freebayes ss somaticsniper mutect2 lancet	intersection	3397	782	1	2453	0.99872	0.24173	0.38925
somaticsniper strelka2 lancet	intersection	3397	778	1	2457	0.99872	0.24049	0.38764
somaticsniper strelka2 mutect2 lancet	intersection	3397	778	1	2457	0.99872	0.24049	0.38764
freebayes ss somaticsniper strelka2 lancet	intersection	3397	774	1	2461	0.99871	0.23926	0.38603
freebayes ss somaticsniper strelka2 mutect2 lancet	intersection	3397	774	1	2461	0.99871	0.23926	0.38603
somaticsniper varscan2 lancet	intersection	3397	771	0	2464	1	0.23833	0.38492
somaticsniper strelka2 varscan2 lancet	intersection	3397	771	0	2464	1	0.23833	0.38492
somaticsniper mutect2 varscan2 lancet	intersection	3397	771	0	2464	1	0.23833	0.38492
somaticsniper strelka2 mutect2 varscan2 lancet	intersection	3397	771	0	2464	1	0.23833	0.38492
freebayes ss somaticsniper varscan2 lancet	intersection	3397	767	0	2468	1	0.23709	0.38331
freebayes ss somaticsniper strelka2 varscan2 lancet	intersection	3397	767	0	2468	1	0.23709	0.38331
freebayes ss somaticsniper mutect2 varscan2 lancet	intersection	3397	767	0	2468	1	0.23709	0.38331
freebayes ss somaticsniper strelka2 mutect2 varscan2 lancet	intersection	3397	767	0	2468	1	0.23709	0.38331
vardict lancet	intersection	3397	753	107	2482	0.87558	0.23277	0.36777
vardict mutect2 lancet	intersection	3397	746	18	2489	0.97644	0.2306	0.37309
vardict strelka2 lancet	intersection	3397	731	28	2504	0.96311	0.22597	0.36605
vardict strelka2 mutect2 lancet	intersection	3397	729	12	2506	0.98381	0.22535	0.3667

vardict freebayes ss lancet	intersection	3397	705	4	2530	0.99436	0.21793	0.35751
vardict freebayes ss mutect2 lancet	intersection	3397	704	2	2531	0.99717	0.21762	0.35727
vardict freebayes ss strelka2 lancet	intersection	3397	694	2	2541	0.99713	0.21453	0.35309
vardict freebayes ss strelka2 mutect2 lancet	intersection	3397	694	2	2541	0.99713	0.21453	0.35309
vardict varscan2 lancet	intersection	3397	683	1	2552	0.99854	0.21113	0.34856
vardict strelka2 varscan2 lancet	intersection	3397	683	1	2552	0.99854	0.21113	0.34856
vardict mutect2 varscan2 lancet	intersection	3397	683	1	2552	0.99854	0.21113	0.34856
vardict strelka2 mutect2 varscan2 lancet	intersection	3397	683	1	2552	0.99854	0.21113	0.34856
vardict freebayes ss varscan2 lancet	intersection	3397	667	0	2568	1	0.20618	0.34188
vardict freebayes ss strelka2 varscan2 lancet	intersection	3397	667	0	2568	1	0.20618	0.34188
vardict freebayes ss mutect2 varscan2 lancet	intersection	3397	667	0	2568	1	0.20618	0.34188
vardict freebayes ss strelka2 mutect2 varscan2 lancet	intersection	3397	667	0	2568	1	0.20618	0.34188
vardict somaticsniper lancet	intersection	3397	520	1	2715	0.99808	0.16074	0.27689
vardict somaticsniper mutect2 lancet	intersection	3397	520	1	2715	0.99808	0.16074	0.27689
vardict freebayes ss somaticsniper lancet	intersection	3397	517	1	2718	0.99807	0.15981	0.27551
vardict freebayes ss somaticsniper mutect2 lancet	intersection	3397	517	1	2718	0.99807	0.15981	0.27551
vardict somaticsniper strelka2 lancet	intersection	3397	513	1	2722	0.99805	0.15858	0.27367
vardict somaticsniper strelka2 mutect2 lancet	intersection	3397	513	1	2722	0.99805	0.15858	0.27367
vardict freebayes ss somaticsniper strelka2 lancet	intersection	3397	510	1	2725	0.99804	0.15765	0.27229
vardict freebayes ss somaticsniper strelka2 mutect2 lancet	intersection	3397	510	1	2725	0.99804	0.15765	0.27229
vardict somaticsniper varscan2 lancet	intersection	3397	508	0	2727	1	0.15703	0.27144
vardict somaticsniper strelka2 varscan2 lancet	intersection	3397	508	0	2727	1	0.15703	0.27144
vardict somaticsniper mutect2 varscan2 lancet	intersection	3397	508	0	2727	1	0.15703	0.27144
vardict somaticsniper strelka2 mutect2 varscan2 lancet	intersection	3397	508	0	2727	1	0.15703	0.27144
vardict freebayes ss somaticsniper varscan2 lancet	intersection	3397	505	0	2730	1	0.15611	0.27005
vardict freebayes ss somaticsniper strelka2 varscan2 lancet	intersection	3397	505	0	2730	1	0.15611	0.27005
vardict freebayes ss somaticsniper mutect2 varscan2 lancet	intersection	3397	505	0	2730	1	0.15611	0.27005
vardict freebayes ss somaticsniper strelka2 mutect2 varscan2 lancet	intersection	3397	505	0	2730	1	0.15611	0.27005

Table S1. **Benchmark of all the combinations of the 7 tools.** The results of all possible combinations of the tested 7 somatic variant callers, including intersection and unions of the considered algorithms.
